# Anti-Hypotensive Treatment and Endothelin Blockade Synergistically Antagonize Exercise Fatigue in Rats under Simulated High Altitude

**DOI:** 10.1371/journal.pone.0099309

**Published:** 2014-06-24

**Authors:** Daniel Radiloff, Yulin Zhao, Alina Boico, Gert Blueschke, Gregory Palmer, Andrew Fontanella, Mark Dewhirst, Claude A. Piantadosi, Robert Noveck, David Irwin, Karyn Hamilton, Bruce Klitzman, Thies Schroeder

**Affiliations:** 1 Department of Radiation Oncology, Duke University Medical Center, Durham, North Carolina, United States of America; 2 Department of Surgery, Duke University Medical Center, Durham, North Carolina, United States of America; 3 Department of Medicine-Pulmonary, Duke University Medical Center, Durham, North Carolina, United States of America; 4 Department of Medicine-Clinical Pharmacology, Duke University Medical Center, Durham, North Carolina, United States of America; 5 Department of Cardiology, University of Colorado Denver, Aurora, Colorado, United States of America; 6 Department of Health and Exercise Science, Colorado State University, Fort Collins, Colorado, United States of America; 7 Department of Physical Chemistry, University of Mainz, Mainz, Germany; Universidad Pablo de Olavide, Centro Andaluz de Biología del Desarrollo-CSIC, Spain

## Abstract

Rapid ascent to high altitude causes illness and fatigue, and there is a demand for effective acute treatments to alleviate such effects. We hypothesized that increased oxygen delivery to the tissue using a combination of a hypertensive agent and an endothelin receptor A antagonist drugs would limit exercise-induced fatigue at simulated high altitude. Our data showed that the combination of 0.1 mg/kg ambrisentan with either 20 mg/kg ephedrine or 10 mg/kg methylphenidate significantly improved exercise duration in rats at simulated altitude of 4,267 m, whereas the individual compounds did not. In normoxic, anesthetized rats, ephedrine alone and in combination with ambrisentan increased heart rate, peripheral blood flow, carotid and pulmonary arterial pressures, breathing rate, and vastus lateralis muscle oxygenation, but under inspired hypoxia, only the combination treatment significantly enhanced muscle oxygenation. Our results suggest that sympathomimetic agents combined with endothelin-A receptor blockers offset altitude-induced fatigue in rats by synergistically increasing the delivery rate of oxygen to hypoxic muscle by concomitantly augmenting perfusion pressure and improving capillary conductance in the skeletal muscle. Our findings might therefore serve as a basis to develop an effective treatment to prevent high-altitude illness and fatigue in humans.

## Introduction

A reduction in physical performance capacity and untimely fatigue are among the deleterious acute effects of rapid ascent to high altitudes. The most important factor underlying altitude-induced fatigue is thought to be the decreased availability of oxygen in arterial blood [1], [2]. Abundant experimental work has been conducted to identify mechanisms to augment arterial oxygen content (C_aO2_) by inducing hematopoiesis and increasing the hematocrit to improve exercise capacity at high altitude, but with variable success [3]–[5]. A more promising approach to improve maximum exercise capacity has been to target the pulmonary circulation, e.g. using dexamethasone, sildenafil, or endothelin blockers. The beneficial effects observed for these drugs have been attributed to a reduction in pulmonary arterial pressure, and/or an improved ventilation-perfusion-matching (V/Q) [6]–[9].

Because high altitude compromises function in multiple organs rather than impacting only the lung, we have hypothesized that a combination of targeting agents, rather than monotherapy approach, has the highest potential to effectively counteract altitude-induced fatigue. Indeed, our group showed previously that the combined dosing with theophylline and the endothelin receptor blocker sitaxsentan significantly increased exercise capacity of rats under simulated high altitude, whereas the single compounds did not [10]. The underlying mechanism appeared to be increased muscle tissue oxygenation via an increased rate of oxygen delivery, rather than by means of increased arterial oxygen content. Our data indicated that the mechanism of action involved a theophylline-induced increase in perfusion pressure on the skeletal muscle, caused by an increase in arterial blood pressure. However, theophylline is a highly pleiotropic drug with both cardiostimulatory and vasodilatory properties, and it has remained unclear whether augmentation of arterial blood pressure was essential for the observed ergogenic effects of the combination treatment.

Vasodilation, particularly of pulmonary vasculature, is a favored drug effect in altitude medicine, mostly because pulmonary vasoconstriction is thought to contribute to high altitude induced pulmonary edema (HAPE) [11]. It is however important to note that systemic hypoxia also produces peripheral arterial vasodilation, which has a profound impact on heart rate, peripheral blood flow, and the ability to compensate for orthostatic challenges [12]–[14]. Thus, some degree of localized vasoconstriction and increased blood pressure may be desirable under these conditions. Because hypertensive treatment heightens pulmonary arterial pressure, and thus potentially increases the risk of HAPE, such interventions would be viewed skeptically by researchers in the field [11], [15]. In the light of the potential benefit of cardiostimulatory treatment, and with respect to our previous work, it is thus important to know whether distinctly hypertensive drug effects as part of a drug combination carry utility to alleviate altitude-induced performance loss.

This study was designed to understand whether the hypertensive drugs ephedrine and methylphenidate would synergize with an endothelin receptor blocker to increase exercise capacity in rats under simulated high altitude. Ephedrine is a natural compound that together with its stereoisomer pseudoephedrine, has seen widespread use as a decongestant and cough suppressant, anti-hypotensive agent, and as a weight-loss supplement [16]–[18]. Methylphenidate is a synthetic amphetamine derivative that has been used to treat hyperactivity and attention deficit disorders [19]. Hypertension is a known effect of both of these drugs, and neither has been previously reported for their potential to mitigate altitude-related performance decrements. We hypothesized that the hypertensive drugs ephedrine or methylphenidate, when combined with an endothelin-1 blocking agent, would increase exercise performance under simulated high altitude in a rat model, whereas the single compounds would not. The proposed mechanism would involve an increased perfusion pressure in peripheral organs such as skeletal muscle, synergizing with a reduction of endothelin-mediated pre-capillary arteriolar vasoconstriction and leading to improved capillary flow and oxygen transport.

## Materials and Methods

### Drug and dosing regimen

All animal procedures were pre-approved by Duke University Institutional Animal Care and Use Committee (DUIACUC). All drugs were administered intraperitoneally (IP). Individual sympathomimetic treatments were [low dose ephedrine (2 mg/kg body wt.); high dose ephedrine (20 mg/kg)], [low dose methylphenidate (4 mg/kg); and high dose methylphenidate (10 mg/kg)]. Combination treatments were ambrisentan 0.1 mg/kg with (1) ephedrine 2 mg/kg (combination ephedrine low), (2) ephedrine 20 mg/kg (combination ephedrine high), (3) methylphenidate 4 mg/kg (combination methylphenidate low), (4) methylphenidate 10 mg/kg (combination methylphenidate high). Normal saline (0.9% NaCl) served as a control.

### Run to fatigue measurements

The protocol to measure the influence of intraperitoneally administered drugs on the exercise capacity of hypoxic rats in a forced rodent exercise wheel system has been published in detail [10]. Briefly, rats were habituated to running in a motorized wheel system (Lafayette Instruments, Lafayette, IN) at 10 min/day for approximately 10 days, and then subjected to exercise testing in the same wheels in a hypobaric chamber at the Duke University Center for Hyperbaric Medicine and Environmental Physiology. The chosen level of simulated high altitude of 4267 m has been shown to strongly decrease voluntary performance in the rat [10]. Treatment agents were injected at near sea level altitude (119 m) 30 minutes before starting the exercise protocol, and the rats were taken to altitude 15 minutes before starting the run to allow for 5–10 minutes of equilibration time at altitude before exercise. An investigator accompanied the rats in the chamber to monitor their performance. The exercise protocol consisted of 10 minutes running at 6 meters per minute (m/min), 80 min running at 9 m/min, and 30 min running at 12 m/min. Up to 15 rats were exercised at the same time in individual wheels. Rats showing signs of fatigue were removed and tested for exhaustion by observing self-locomotion on a flat surface for 30 seconds. Rats that continued to show failure to run on the wheel, or acquired injuries during the run were immediately removed from the experiment, and treated as “censored” in log-rank tests.

### Hemodynamic measurements on anesthetized animals

Experiments for the measurements of heart rates, mean arterial pressure, pulmonary arterial pressure, and arterial hemoglobin saturation were designed so that the dosing schedules would mirror the sequence of events and timing of the run-to-fatigue trials. Rats were anesthetized with ketamine/xylazine (80 mg/kg/8 mg/kg), and placed in lateral recumbency on a heating pad, to maintain body temperature. Indwelling PE-50 catheters were placed in the carotid artery for measurements of mean arterial blood pressure and PV-1 catheters were inserted into the pulmonary artery, via the jugular vein, to measure pulmonary arterial pressure as described [10], [20]. Collection of arterial pressure data via a fluid-filled pressure transducer as was described [10]. The experimental protocol consisted of 15 min at normoxia (oxygen/nitrogen mixture to maintain 93–96% arterial HbO_2_, typically 30%), followed by drug injection and after another 15 min, by inspired hypoxia (12%, balance nitrogen) for at least 30 min more. Gas mixtures were administered at constant flow rate of 2.5 l/min using a manual adjustment system (Oxydial, StarrLife Sciences, Oakmont, PA) Breathing rates were measured by counting chest movements over successive time periods from video sequences: directly before drug injection (min 10–15), directly before onset of hypoxia (min 25–30), and under hypoxia (min 50–55). Heart rates and arterial hemoglobin saturation were measured using pulse oximetry on the hind paw (MouseOx, StarrLife Sciences, Oakmont, PA).

### Laser Doppler blood flow measurements

Continuous hind-limb blood flow measurements were performed on ketamine/xylazine anesthetized rats using laser Doppler probes (Oxyflow, Oxford Optronix, UK) placed directly on the vastus lateralis muscle with the overlying skin removed.

### Tissue pO_2_ measurements

Tissue pO_2_ measurements were performed using invasive needle-encased probes (Oxylite, Oxford Optronix, UK) inserted into the vastus lateralis muscle parallel to the muscle fibers, with overlying skin removed. Because the amplitude of response of inserted probes to changes in inspired oxygen is partially dependent on the placement of the probe, we added a pre-treatment cycle of hypoxia/normoxia, as outlined previously, to control for differences in probe placement [10]. In brief, animals were subjected to baseline normoxia (sufficient oxygen to yield 93–96% HbO_2_) for at least 10 min, then hypoxia (12%) for 15 min, then normoxia for 10 min, followed by the treatment drug, followed by the above schedule for anesthetized rats, i.e. 15 min normoxia, and 30 min hypoxia. Because the experiment required that the needle probe be placed in a way that it responds to physiological changes in tissue O_2_ delivery, experiments where pre-treatment muscle pO_2_ recordings did not respond to inspired hypoxia were eliminated from the study.

### Pulmonary flow measurements

Blood flow velocity in the pulmonary microcirculation was measured via intravital microscopy on pentobarbital-anesthetized, ventilated rats (50 mg/kg), after injection of fluorescently labeled blood cells, using a thoracic window as described [21]. A different anesthesia regimen was necessary for this experiment in order to eliminate autonomous breathing. Measurements were performed with a CCD camera (Andor, Belfast, UK), and blood flow velocity was quantified using a Matlab-based computer algorithm. [21]. The experimental schedule was the following: 15 min baseline, first injection of ephedrine/ambrisentan at 20/0.1 mg/kg, after 15 min injection of a second dose of ephedrine/ambrisentan (same dose), end of experiment 15 min thereafter. Imaging under apnea was performed every 5 minutes. Animals were ventilated on a mechanical ventilator via a tracheal catheter and received sufficient oxygen to maintain HbO_2_ at 93–96% HbO_2_ (typically 30% FiO_2_).

### Statistics

Comparisons between run-to-fatigue curves were performed using log rank tests, and correction for multiple comparisons was conducted by multiplying the resulting p values by the number of comparisons. Animals that had to be removed from the exercise trials prior to fatigue, e.g. because of injury, were treated as “censored”, i.e. their running history was only considered in the analysis until the time the injury became apparent. Comparisons between multiple data sets were conducted using one-way ANOVA for independent variables, and repeated-measures ANOVA for linked variables. All ANOVA analyses were corrected for multiple comparisons by the Bonferroni method. Continuous variables between two groups were compared using either T-test, or nonparametric Mann Whitney U-test, depending on whether the data was assumed to follow a normal Gaussian distribution, or not. GraphPad Prism software was used throughout to analyze the data sets (Graphpad, San Diego, CA). In the figures, single asterisks (*) represent p-values of less than 0.05, whereas double asterisks (**) represent p-values of less than 0.01. All error bars and parameters of variation in the text represent the standard deviation.

## Results

### Run to fatigue

**Figure 1:**
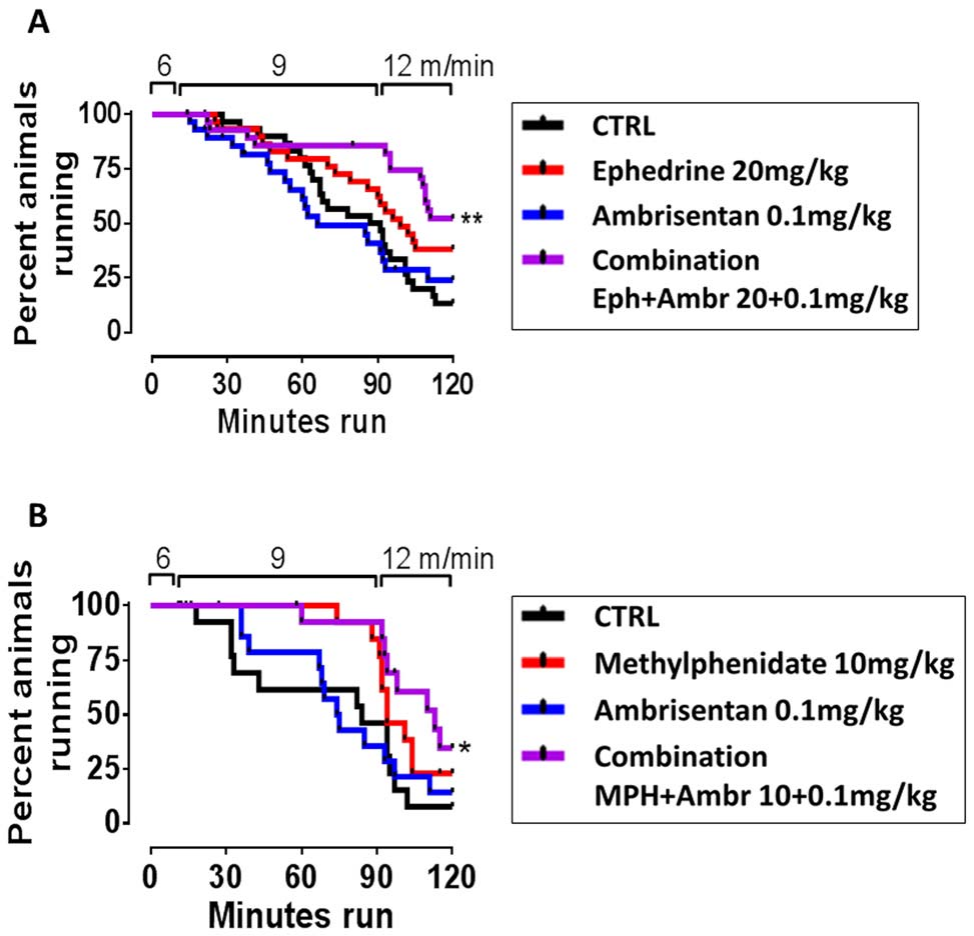
A: The combination of ephedrine (20 mg/kg) and ambrisentan (0.1 mg/kg), but not the single compounds, significantly increase the time run to fatigue of rats under simulated high altitude (Log rank test p = 0.0024 after correction for multiple comparison). Lower concentrations of ephedrine (2 mg/kg) were not ergogenic, neither alone nor if combined with ambrisentan (not shown). N = 28–31 per treatment group. B: Methylphenidate (10 mg/kg) combined with ambrisentan, but not the single compounds, enhanced time run to fatigue under simulated high altitude (Log Rank test p = 0.044 after correction for multiple comparison). Lower dose methylphenidate (4 mg/kg) did not have ergogenic effects, alone or in combination with ambrisentan (not shown). N = 14–15 per treatment group. N = 10–11 per treatment group. All drugs were injected IP.

In our escalating exercise protocol under simulated high altitude, only rats treated with a combination of a high dose sympathomimetic (ephedrine 20 mg/kg or methylphenidate 10 mg/kg), and ambrisentan (0.1 mg/kg), ran significantly longer than control rats. Use of single compounds or low dose sympathomimetics (ephedrine 2 mg/k and methylphenidate 4 mg/kg, Figure 1A, B) did not improve exercise performance beyond that of controls.

### Heart rates

**Figure 2:**
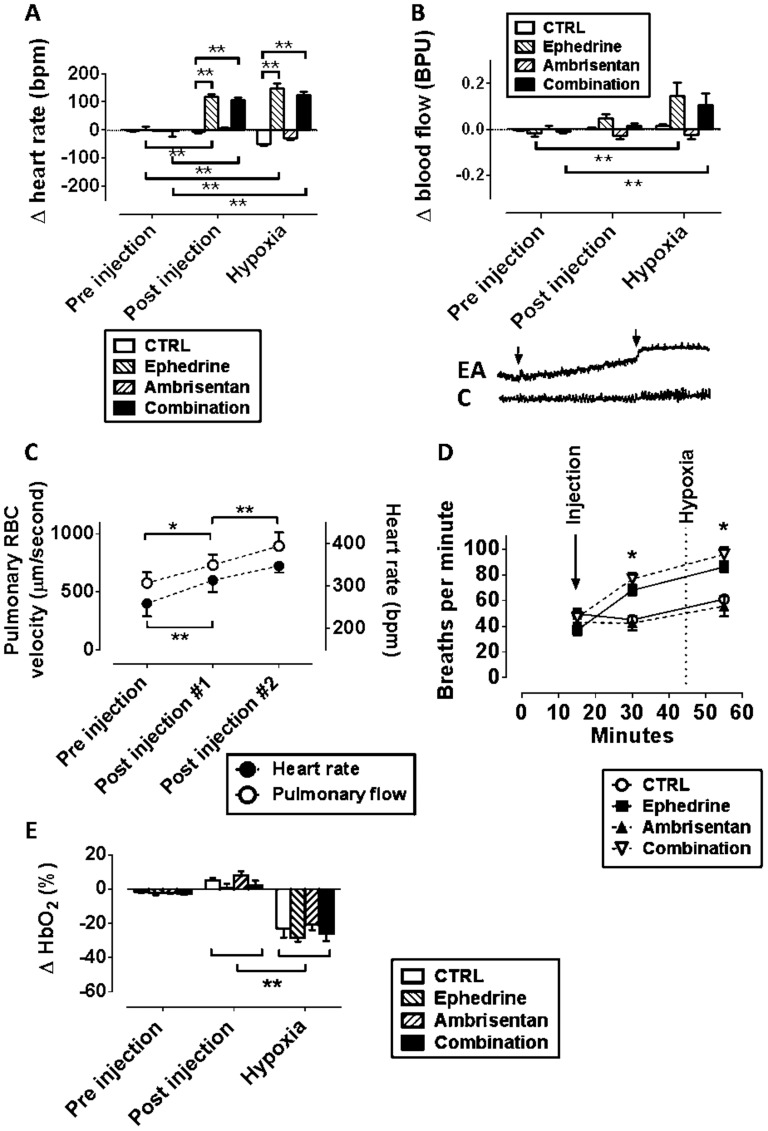
Hemodynamic effects of high-dose ephedrine in anesthetized rats. “Pre-injection” were averaged data −5 to 0 minutes pre injection, “post injection” was averaged −5 to 0 minutes before onset of hypoxia, and “hypoxia” was averaged 30–40 minutes post injection. A: Changes in heart rates, normalized to the time of injection. The addition of ephedrine at 20 mg/kg IP significantly increased heart rates, alone and combined with ambrisentan. Heart rates remained significantly elevated under hypoxia in ephedrine treated groups (one-way ANOVA/Bonferroni, p<0.01, N = 7–13). B: Changes in blood perfusion velocity in hind leg muscle after treatment, measured directly on the muscle, using a laser Doppler probe. Both ephedrine and the combination significantly enhanced muscle blood flow under hypoxia (N = 6–9). The onset of hypoxia triggered a distinct increase in muscle flow in all groups containing ephedrine, and to a lesser degree in control treated animals (example laser Doppler tracings from rat leg muscle; EA = ephedrine and ambrisentan; C = combination; first arrow: injection, second: hypoxia). C: Repeated injection of combined ephedrine (20 mg/kg) and ambrisentan (0.1 mg/kg) significantly enhances heart rates, and concomitantly, pulmonary blood flow, measured by pulmonary window blood flow measurements, and pulse oximetry (paired T-test, corrected for multiple comparison, p<0.05, N = 5). D: injection of ephedrine, alone or in combination with ambrisentan, caused a significant increase of breathing rates (one-sided ANOVA with Bonferroni correction, p<0.001, N = 5–13/group). Hypoxia further increased breathing rates in all groups (repeated measures ANOVA/Bonferroni, p<0.001). E: Changes in blood oxygenation after treatment: HbO_2_ decreased significantly in all treatment groups after onset of inspired hypoxia (repeated measure ANOVA/Bonferroni, p<0.001), but there was no difference in this parameter between treatment groups (one-way ANOVA/Bonferroni, N = 5–9).

Mean heart rates from pooled data from all treatment groups at baseline were 283.2±47.8 beats per minute (BPM). Heart rates increased significantly after treatment with ephedrine or combination, and remained elevated under hypoxia (comparison between time points for each treatment: repeated measures ANOVA with Bonferroni correction, all p<0.01. Comparison between treatment groups by one-way ANOVA with Bonferroni correction, all p<0.01, Figure 2A).

### Blood flow in hind leg muscle

Under hypoxia, both ephedrine and combination-treated animals displayed significant increased blood flow in the vastus lateralis muscle in the hind leg, compared to control and ambrisentan only treated animals (Figure 2B). Hypoxia triggered an immediate increase in the muscle blood flow tracings in most animals in treatment groups; however, when comparing flow 1 min pre- vs. 1 min post-injection of treatment, no significance was found in any of the groups (Wilcoxon Rank test for repeated measures).

### Pulmonary capillary blood flow

Treatments with combined ephedrine and ambrisentan sequentially increased blood flow velocity through the pulmonary capillary system, measured with pulmonary intravital microscopy. This increase was due mainly to an increase in heart rates (Figure 2C). Treatment with saline did not lead to a comparable change (data not shown).

### Breathing rates

Treatment with ephedrine (20 mg/kg), alone and in combination, significantly increased breathing rates compared to groups without ephedrine. Hypoxia further increased breathing rates in all groups (Figure 2D).

### Arterial blood oxygenation

Mean hemoglobin oxygen saturation (HbO_2_) from pooled data of all treatment groups at baseline was 97.3±4%. All treatment groups experienced a significant decrease in HbO_2_ after onset of hypoxia, but no differences in HbO_2_ were detected among the treatment groups (Figure 2E).

### Mean arterial pressure (MAP)

**Figure 3:**
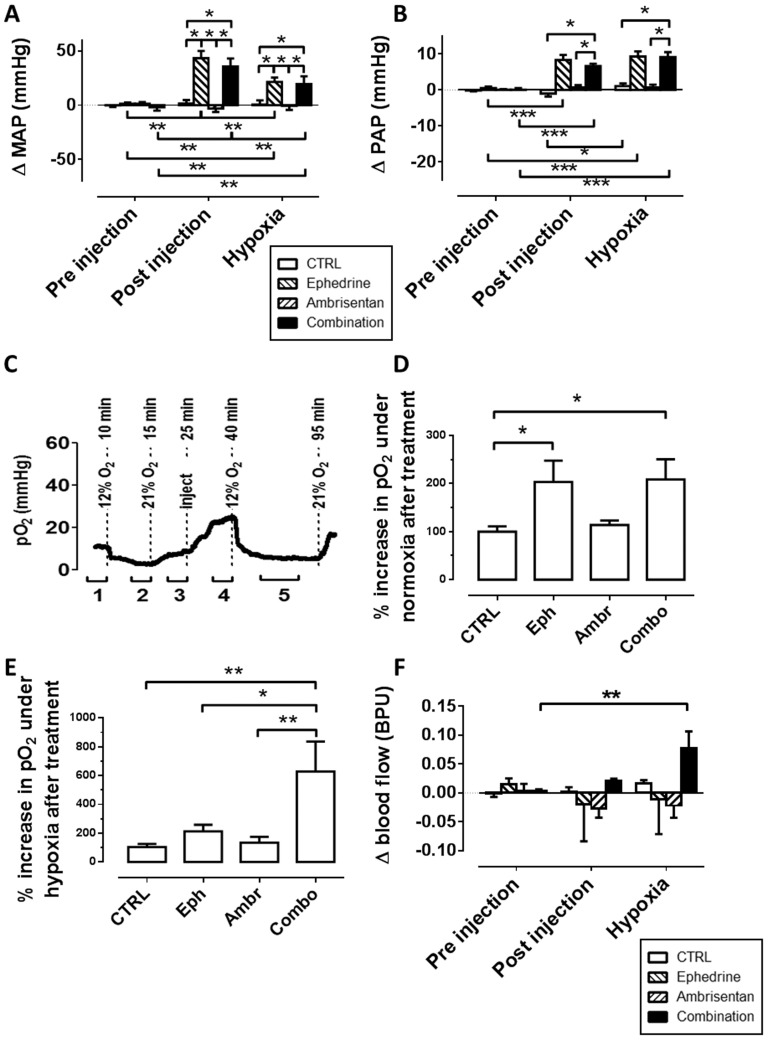
Blood pressure effects of ephedrine treatment. “Pre-injection” were averaged data −5 to 0 minutes pre injection, “post injection” was averaged −5 to 0 minutes before onset of hypoxia, and “hypoxia” was averaged 30–40 minutes post injection. A, B: Treatment with high dose ephedrine (20 mg/kg), alone and combination with ambrisentan, significantly increased MAP and PAP, under normoxia and hypoxia, compared to baseline (repeated measures ANOVA with Bonferroni correction, p<0.05), and compared to the other treatment groups (one-way ANOVA/Bonferroni, p<0.001). Onset of hypoxia significantly decreased MAP in these groups (repeated measures ANOVA with Bonferroni correction, p<0.05). In the control group, PAP increased significantly after onset of hypoxia (N = 7–14). C: Experimental schedule to measure the influence of treatment on oxygen concentration in the hind muscle. Tissue hypoxia was measured by pO_2_ needle electrode tracings during inspired normoxia (1) and hypoxia at baseline (2), before (3) and after injection under normoxia (4), and during post-injection hypoxia (5). D: under post-treatment normoxia (panel D time point #4), ephedrine alone and in combination with ambrisentan significantly increased muscle oxygenation, compared to pre-treatment pO_2_ (one-way ANOVA with Bonferroni correction, N = 4–8, p<0.05). E: Under post-treatment hypoxia (panel D #5), only the combination of high ephedrine and ambrisentan significantly increased muscle oxygen tension. Re-oxygenation after combo treatment was significantly higher than in all other treatment groups (one-way ANOVA/Bonferroni, N = 4–8, p<0.05 * or 0.01**). F: Low dose ephedrine (2 mg/kg) significantly increases blood flow in the hind leg muscle when combined with ambrisentan, but not alone.

Mean MAP in pooled data of all treatment groups at baseline was 90.5±15.2 mmHg. Increases in MAP after treatment and under hypoxia, compared to baseline, were significant in all groups containing ephedrine. Blood pressures in ephedrine and combination treated groups were significantly higher than controls and ambrisentan only, respectively, at each time point post treatment (Figure 3A).

### Pulmonary arterial pressure (PAP)

Mean PAP in pooled data of all treatment groups at baseline was 12.3±5.3 mmHg. Increases in PAP after ephedrine containing treatments were significant both after treatment, and under subsequent hypoxia, compared to baseline. Also, PAP was significantly elevated compared to both controls and ambrisentan at all post treatment time points (Figure 3B).

### Tissue oxygenation

The influence of treatment with ephedrine, ambrisentan, and the combinations was tested by comparing tissue pO_2_ in the rat hind leg vastus lateralis muscle under inspired hypoxia (12% O_2_) before and after injection. The pO_2_ measurements were integrated over (1) the last 5 min before onset of initial hypoxia, (2) the last 5 min before restoration of normoxia, (3) the last 5 min before injection of treatment, (4) the last 5 min before onset of hypoxia, and (5) min 30–40 post injection, i.e. during hypoxia (Figure 3C, example pO_2_ tracing). Average pO_2_ values ± standard deviation for time points 1–5 are given in Table 1. Treatment effects on the tissue oxygen levels under (pre-hypoxic) normoxia were measured as the percentage increase in pO_2_ from time point (3) to time point (4). The ability of the treatment to increase tissue pO_2_ under hypoxia was measured as the percentage increase from time point (2) to time point (5). Under post-treatment normoxia, both ephedrine and ephedrine combined with ambrisentan significantly increased muscle tissue oxygenation, when compared with control treatments (Figure 3D). Under post-treatment hypoxia, only the combination of high dose ephedrine significantly increased muscle oxygenation over all other treatment groups (Figure 3E).

**Table 1:** Averaged tissue pO_2_ values in mmHg (means ± SD), acquired with Oxylite probes.

	*Tissue pO_2_ (mmHg)*				
Time points	1	2	3	4	5
**Control**	14.7±10.3	6.1±5.7	10.5±7.6	10.5±11	5.2±6.3
**Ephedrine low (2 mg/kg)**	20.5±9.5	7.1±4.3	17.6±12.2	19.2±11.7	9.6±5.6
**Ephedrine high (20 mg/kg)**	16±12.8	7±7	21.5±9.5	30.2±19.7	10.8±9.5
**Ambrisentan (0.1 mg/kg)**	19.1±9.4	3.9±1.7	8.1±4.1	9.6±6.3	4.2±2
**Combination Ambr.+Eph. Low**	14.7±4.3	3.5±1.4	8.4±2.7	17.8±10.6	8.7±4.5
**Combination Ambr.+Eph. High**	14±11.3	4±3.7	17.9±10.1	35.3±23.1	15.7±10.9

*Time point 1: averaged over last 5 min before onset of 1^st^ cycle of hypoxia; time point 2: averaged over last 5 min before return to normoxia; time point 3: averaged over last 5 min before injection; time point 4: averaged over last 5 min before onset of 2^nd^ cycle of hypoxia; time point 3: averaged over 30–40 minutes post injection, i.e. during post-treatment hypoxia; Ambr = Ambrisentan, Eph = Ephedrine.*

### Hemodynamic effects of low-dose ephedrine

Experiments were repeated in the ephedrine treatment groups at a 10-fold lower dose of ephedrine, i.e. 2 mg/kg. Both treatments, ephedrine alone and in combination with ambrisentan, significantly increased the heart rate by approximately 45 bpm, compared to baseline (repeated-measures ANOVA/Bonferroni, p<0.05). Blood flow in hind leg muscle was significantly elevated under hypoxia following treatment with the combination, but not with the single compounds (Figure 3F). Breathing rates, HbO_2_, MAP and PAP were unaffected by treatment with the reduced ephedrine dose, alone and in combination with ambrisentan (data not shown).

## Discussion

We tested the novel hypothesis that combined dosing with a chronotropic, hypertensive drug and the endothelin receptor antagonist ambrisentan, under simulated high altitude, would produce a more distinct ergogenic effect than the single compounds alone. Indeed, rats that were dosed with the combination of ephedrine or methylphenidate and the endothelin receptor antagonist (ERA) ambrisentan ran significantly longer than controls under simulated high altitude, whereas those treated with single drugs did not. In anesthetized rats, both ephedrine and ephedrine combined with ambrisentan increased heart rates, MAP, PAP, breathing rates, blood flow to the hind limb musculature, and normoxic oxygenation, but only the drug combination significantly increased muscle oxygenation in hypoxic air.

Several compounds have been suggested for the potential alleviation of altitude-induced fatigue, including inhibitors of phosphodiesterase type 5 (PDE5), dexamethasone, endothelin receptor antagonists, and erythropoietin (EPO) pre-treatment [3], [6]–[8]. Of note, increasing hematocrit by “blood doping” has not been demonstrated to enhance performance capacity at high altitude [5]. We recently reported that the combination of theophylline and the endothelin receptor antagonist (ERA) sitaxsentan synergized to improve exercise performance of rats under hypobaric hypoxia, exceeding the effects of theophylline or sitaxsentan given alone [10]. In addition, we showed that the improved exercise performance was due to increased blood flow (thus, oxygen transport rate) to peripheral tissue, potentially driven and powered by the augmenting effect of theophylline on heart rate and arterial blood pressures [10]. Subsequently, we have here investigated the effects of pharmacologically-induced hypertension in combination with endothelin blockade, using a rodent exercise model under simulated high altitude.

### Study drugs

Ephedrine is a naturally-occurring sympathomimetic amine that shares structural and functional similarity with amphetamine, as well as with the neurotransmitter epinephrine [22]. Ephedrine directly activates alpha- and beta-adrenergic receptors, triggers catecholamine release, and inhibits norepinephrine reuptake [23], [24]. Physiologically, ephedrine increases heart rate, peripheral resistance and arterial pressure, and may cause hallucinogenic side effects [25]. In addition to its medical use, ephedrine has been tried for many years as a performance-enhancing agent; however, consistent ergogenic effects are only achieved if combined with agents such as caffeine [26]. At high doses, ephedrine can produce cardiotoxicity, especially when taken together with caffeine [23], [27], [28]. In rats, ephedrine doses of 20 mg/kg i.p. (12.1% of the intraperitoneal LD_50_ of ephedrine in rats) increases locomotion, which provided the basis for the dosage used in this study [29], [30].

Methylphenidate (MPH) is a benzylpiperidine derivative, structurally related to amphetamine, used for treating attention deficit disorder and depression [19]. Its CNS stimulant activity is mediated through re-uptake inhibition of norepinephrine and dopamine. Methylphenidate also increases norepinephrine levels in the blood, and thus raises heart rate and blood pressure [31]. MPH has shown efficacy as a single-drug performance enhancing agent in normobaric human subjects [32].

Ambrisentan is a competitive antagonist of type A receptors (Et_A_) of the vasoconstrictive peptide hormone endothelin-1 [33]. Endothelin-1 is a dominant factor in tissue blood flow regulation and particularly important in regulating pre-capillary arteriolar tone [34]–[37]. Endothelin receptor antagonists (ERAs), such as ambrisentan, are used to treat pulmonary arterial hypertension in humans [33], [38]. Drug class toxicity of ERAs include liver injury and birth defects, however, ambrisentan has been shown to be relatively safe [38], [39]. More recent research has shown that blockade of endothelin-1 receptors may improve maximum exercise performance at altitude in humans [8], [40]. The dose of ambrisentan selected here was based on our previous research and is lower than that of most other studies in rats [41]. After oral dosing, ambrisentan has a plasma peak at approximately two hours and an elimination half-life of approximately 15 hours [33], [42]. Because ambrisentan has little effect on hepatic cytochrome P_450_ enzymes, its potential to interact with the other drugs given here is modest [33].

### Exercise model and environmental conditions

We utilized an established model of escalated exercise performance in rats at altitude based on motorized rodent wheels [10]. The chosen simulated altitude level of 4267 m is sufficient to decrease voluntary exercise performance by over 60% in rats [10]. While this model is well-suited to read out exercise performance capacity under the given altitude conditions, it is technically not feasible to use it to quantify exercise performance under normoxia, because of the excessive time necessary to produce fatigue in normoxic rats, which causes early termination due to injury before fatigue (data not shown).

### The use of anesthetized animals for mechanistic studies

Although it is possible to perform hemodynamic measurements on conscious rats, reliable needle-based pO_2_ measurements in tissues, which are essential for this study, are not feasible in those models. We have selected anesthetic regimen that are known, and confirmed by our data, to have a comparably small effect on the cardiovascular system, as ketamine/xylazine does not affect the heart rate and only slightly reduce mean arterial pressure at higher doses [43]–[45]. While it is clear that cardiovascular differences between anesthetized resting and awake exercising animals are drastic, effects of hypoxia on the organism, such as hypoxemia, and hypoxic pulmonary vasoconstriction, and sympathetic activation are found in both states [10], [45]. This is demonstrated e.g. by the expected increase of PAP after onset of hypoxia in control animals (Figure 3B).

### Synergism between hypertensive drugs and endothelin blockade under hypoxia

The combination of ephedrine (20 mg/kg) and ambrisentan, as well as ephedrine alone, increased arterial blood pressure in anesthetized rats, both treatments also increased muscle blood flow under hypoxia. Our results obtained in the pulmonary window showed that the drug combination can, in principle, lead to increased pulmonary blood flow, driven by augmented cardiac output. Thus, our data support that the known ability of ephedrine to increase peripheral muscle blood flow is not impaired by the addition of ambrisentan and that the treatment also has the potential to improve pulmonary blood flow.

In agreement with the finding of peripheral blood flow enhancement in anesthetized rats, both ephedrine and its combination with ambrisentan enhanced oxygen delivery to the vastus lateralis muscle under normoxia. Importantly, only the combination, and not ephedrine alone, increased muscle oxygenation under inspired hypoxia. This is in remarkable conformity with the finding that only the combination, but not any of the single compounds, was ergogenic in exercising hypoxic rats.

In mammalian skeletal muscle, autoregulation of blood flow is critically important to the efficient distribution of blood supplied by the systemic vasculature to the region of demand [46], [47]. For example, in resting rats, red skeletal muscle fibers are typically better perfused than white glycolytic fibers, but regional blood supplies are progressively shifted towards glycolytic fibers as maximum exercise intensity is approached [46]. Factors that are known to play a role in the autoregulation of muscle blood flow are e.g. flow/pressure-related effects such as the myogenic response, metabolic vasodilators such as adenosine [48], and local hypoxia [49], [50]. Local hypoxia appears to function as a regional second messenger to redirect organ-specific blood flow to the area of greatest need, and thus, systemic hypoxia drives the fine-tuned local flow regulation in peripheral organs and the brain out of balance [51]–[53]. One of the well-known extrapulmonary cardiovascular effects of hypoxia is NO-mediated peripheral vasodilation, which, after rapid ascension to high altitude, leads to systemic arterial hypotension, and subsequently, to compensatory increases in heart rates [14], [54], [55]. However, hypoxia is also capable of causing peripheral vasoconstriction in resistance arterioles, by triggering the release of the vasoconstrictor endothelin-1 from endothelial cells [56]–[58]. Importantly though, while NO exerts its vasodilatory effect predominantly on 1^st^ and 2^nd^ order arterioles [59], endothelin-1 vasoconstricts only pre-capillary (3^rd^ or higher order) arterioles [37], [60], and, via pericytes, also directly controls capillary diameter [61], [62]. Via endothelin-1 release, hypoxia is therefore capable of directly controlling capillary conductance.

The importance of maintaining perfusion pressure during hypoxic vasodilation to enable efficient capillary blood flow has been recently addressed [49], [50]. In our study, ephedrine and ambrisentan exerted ergogenic effects only when ephedrine concentrations were used at doses that were sufficient to raise pulmonary and mean arterial blood pressure in anesthetized rats. Sympathetic activation, such as triggered by ephedrine or methylphenidate, specifically constricts larger, low-order peripheral arterioles [63]. It is therefore plausible that the sympathomimetic treatment used in this study directly counteracted hypoxia-triggered, NO-mediated vasodilation, thereby reversing arterial hypotension and preserving perfusion pressure on the skeletal muscle. Sympathetic activation and endothelin blockade should therefore synergize to improve capillary perfusion and thus, increase oxygen transport to the hypoxic muscle. Indeed, the observed increase in muscle blood flow and oxygenation in anesthetized rats, in the absence of changes to HbO_2_ suggests that the observed enhancement of exercise performance in awake animals is mediated by changes in flow, rather than by arterial oxygen content (CaO_2_).

The observed increase in ventilation rate after treatment with ephedrine has been reported, but since it had no apparent impact on blood oxygen concentrations, this effect may not carry ergogenic significance.

It is well known that the mammalian cardiovascular system responds to hypoxemia with increased cardiac output, which can, to some degree, be succeeded by increased muscle blood flow [54], [64]–[69]. This mechanism is probably responsible for the immediate, steep increase in muscle blood flow that was seen in many animals, following the onset of hypoxia shown in Figure 2B. Both the apparent extrapulmonary effect of ambrisentan on muscle, and the interaction between pharmaceutically-induced and hypoxia-stimulated increases in peripheral blood flow, will be important future topics for investigation, to further elucidate and leverage this cooperative drug effect.

While we focused on exercise performance as a readout for the detrimental effects of altitude on animals, the combined sympathomimetic/endothelin blocking treatment may also improve other symptoms of altitude sickness. Endothelin-1 has a role in the cerebral microcirculation that is remarkably similar to the muscle, with pre-capillary arterioles being particularly responsive to endothelin-1-mediated vasoconstriction [70], [71]. Endothelin-mediated vasoconstriction in the brain, in principle, can be pharmacologically reversed [72]. It is also known that cerebral blood flow is responsive to hypoxia [52], [73], and that systemic arterial hypotension, as e.g. elicited by hypoxia, will compromise cerebral blood flow if it falls below a critical threshold [74]. While we did not investigate whether endothelin-1 release plays a major role in cerebral pathophysiology at high altitude, it seems possible that combined sympathomimetic/endothelin-1 blockade may also counteract altitude-related central nervous system effects, such as acute mountain sickness (AMS).

Given the toxicity of high-dose ephedrine, it is important to ask whether the effective dose in our study is clinically feasible in humans. While detailed pharmacokinetic data on rats is not available in the literature, there is enough information for a rough prediction of the human equivalent dose (HED) to what we have found to be efficacious in rats. By extrapolation, ephedrine at 20 mg/kg intraperitoneally equals approximately 0.81 mg/kg intravenously [75]. This approximates a plasma concentration of 58.7 ng/ml [76]. Because this is only about one fifth of the human C_max_ of approximately 290 ng/ml measured after ingestion of a moderate single dose of 75 mg [77], the HED of ephedrine is probably in the clinically relevant range. However, cardioactive drugs should be used with great caution under high altitude, especially in the elderly and/or those with cardiopulmonary disease [78], [79]. In addition, the increase of pulmonary arterial pressure caused by ephedrine has the potential to augment pulmonary leak in humans, thus potentially increasing the risk of developing HAPE [80]. However, among the factors known to predispose to HAPE, the exaggerated production of endothelin-1 leads to impaired plasticity and adaptability of the pulmonary vasculature [81]. It must be carefully tested whether the combined use of sympathomimetics and endothelin blockers increases or decreases the risk of HAPE.

In summary, we have presented evidence that the combination of the hypertensive drugs ephedrine or methylphenidate with the endothelin A receptor antagonist ambrisentan increase the exercise capacity of rats under simulated high altitude. The underlying mechanism probably involves a specific increase in capillary blood flow in skeletal muscle, and an elevated arterial blood pressure appears to be critical for this effect. Our data indicate that while ephedrine alone increases muscle oxygenation under normoxia, the combination with ambrisentan specifically re-oxygenates hypoxic muscle, whereas ambrisentan has little or no effect on normoxic muscle. Other hypertensive drugs, such as methylphenidate, demonstrate effects similar to ephedrine when combined with ambrisentan. These data suggest that hypertensive and endothelin-blocking drug combinations could effectively mitigate performance loss during rapid ascent to high altitudes in humans.
